# The effect of psychoeducational intervention, based on a self-regulation model on menstrual distress in adolescents: a protocol of a randomized controlled trial

**DOI:** 10.1186/s13063-020-04629-z

**Published:** 2020-08-27

**Authors:** Somayeh Asgari, Zainab Alimoardi, Mohammad Ali Soleimani, Kelly-Ann Allen, Nasim Bahrami

**Affiliations:** 1 Student Research Committee, Qazvin University of Medical Sciences, Qazvin, Iran; 2 Social Determinants of Health Research Center, Research Institute for Prevention of Non-Communicable Diseases, Qazvin University of Medical Sciences, Shahid Bahonar Blvd, Qazvin, 3419759811 Iran; 3 Educational Psychology and Inclusive Education, Faculty of Education, Monash University and The Centre for Positive Psychology, The Melbourne Graduate School of Education, The University of Melbourne, Melbourne, Australia

**Keywords:** Menstrual distress, Dysmenorrhea, Illness perceptions, Self-regulation model

## Abstract

**Introduction:**

Menstrual distress caused by primary dysmenorrhea is associated with physical and psychological symptoms—before, after, and during menstruation. Leventhal’s self-regulation educational model is based on the cognitive and emotional experiences of threat responses to symptoms and relates to coping responses. This study aims to investigate the effect of the implementation of a psychoeducational intervention, based on the self-regulation model of menstrual distress in adolescents.

**Methods/design:**

In this randomized controlled trial, 120 adolescent girls with moderate to severe menstrual pain (based on visual analog scale (VAS) ≥ 4) from twelve randomly selected high schools in Qazvin City will be enrolled in the study and will be randomly assigned to either a 3-session psychoeducational intervention (*n* = 60) or control (*n* = 60) groups. The sessions will be between 60 and 90 min apiece, and they will run for three consecutive weeks (one session per week). The data collection tools will include questionnaire eliciting menstrual information and demographics, the VAS, the Moos Menstrual Distress Questionnaire, and the illness perception questionnaire. One month prior to the intervention, both groups will participate in an initial assessment to assess the severity of their pain and level of menstrual distress. Finally, all questionnaires will be completed for three consecutive months after the intervention is completed.

**Discussion:**

It is anticipated that findings of this study will provide evidence for the effectiveness of the Leventhal self-regulation model. Implications for improved practice, understanding, and treatment for menstrual distress may also arise.

**Ethical considerations:**

The research protocol will be reviewed by the ethics committee, which is affiliated with the Qazvin University of Medical Sciences (Decree code: IR.QUMS.REC.1398.043).

**Trial registration:**

IRCT20190625044002N1. Registration date: 2019-09-03.

## Background

Dysmenorrhea (i.e., painful menstruation) is one of the most common gynecological problems and is characterized by acute pelvic pain during menstruation [1, 2]. Depending on the pathological and anatomical statuses, dysmenorrhea is divided into two types of primary and secondary dysmenorrhea [3]. Primary dysmenorrhea is the presence of painful menstruation in the absence of pelvic diseases (e.g., endometriosis and pelvic inflammatory disease) or pelvic leiomyoma [4]. The results of a review of 15 primary studies published between 2002 and 2011 showed that the prevalence of primary dysmenorrhea ranged between 16 and 91% in women over the age of 13, and severe dysmenorrhea was reported as being between 2 and 29% [5]. According to a systematic review of Iranian studies (2016), the prevalence of primary dysmenorrhea was 71% [6]. Momenzadeh et al. reported that the prevalence of dysmenorrhea was 52.1% in Iranian girls under 15 [7].

Primary dysmenorrhea is the most common form of chronic pelvic pain that can last for at least 6 months, can be severe enough to disrupt one’s daily life activities, and ultimately lead to medical or surgical treatment. Chronic pelvic pain associated with the patient’s menstrual cycle is referred to as cyclic chronic pelvic pain [3]. Similar to other chronic pains, menstrual pain can act as a stressor and exacerbate psychological distress (e.g., the symptoms of depression and anxiety) [2].

During the management of dysmenorrhea, the relationship between dysmenorrhea and psychological problems are considered by most clinicians [8, 9]. The physical and psychological symptoms of primary dysmenorrhea (i.e., menstrual distress) may occur before, after, and during menstruation [10]. Menstrual distress is one of the most common problems in adolescence, with [11] 75 to 94% of women experiencing the symptoms [12]. Regarding treatment, 58–90% of adult women prefer bed rest as a preventive treatment when they experience menstrual distress [13]. Menstrual distress is generally associated with symptoms such as irritability, tenderness of the breasts, low back pain, skin disorders, fatigue, palpitations, loneliness, nausea, vomiting, abdominal pain, and general weakness [14]. Painful menstruation and menstrual distress are associated with a deterioration of quality of life, as well as negative socioeconomic consequences [7, 15]. About 1% of women over the age of 18 are absent from work 1 to 3 days a month, due to severe dysmenorrhea. About 14% of adolescent girls miss school each month, due to painful menstruation [16, 17]. Given the impact on healthcare, the reduction in school attendance, and potential socioeconomic impacts of menstruation, this is an area that requires more recognition [5].

Despite various treatments for relieving menstrual pain and its associated symptoms, there is a need to find non-pharmacological approaches without causing side effects to adolescents’ general health [4]. Various studies have shown the efficacy of complementary and alternative therapies (e.g., herbal remedies [18–20], exercise [11], heat [21, 22], and hypnosis [23]) at reducing menstrual pain. Cognitive behavioral therapy is one of the non-pharmacological treatments that can effectively relieve dysmenorrhea as psychological factors can exacerbate pain [24].

The Leventhal self-regulation model is used to understand illness perceptions [25] and provides a framework for the organization of individuals’ perceptions about health, illness, and outcomes. This theory describes cognitive processes, emotional responses, and consequences of behaviors associated with health and illness [26]. Illness perceptions include responses to symptoms (e.g., weakness and lethargy) by directing cognitive and emotional experiences to related threats (e.g., dysmenorrhea) and guiding coping responses [27]. The model posits that people’s cognitive perceptions about illness are related to five dimensions, which include beliefs about identity (e.g., related to diagnosis or symptoms of the disorder), causes (e.g., ideas about the origins), consequences (e.g., the impact on different aspects of life), timelines (e.g., thoughts about the duration of the disorder), and treatment or control (e.g., beliefs about the treatment and improvement of the disorder). Emotional perceptions also include negative reactions including fear, anxiety, anger, and discomfort [25].

Illness perceptions can affect health-related behaviors and adaptive behaviors that the patient requires to be able to manage, or cope with, a disease [28]. Research shows that a person’s understanding of a disease is an important determinant of self-care [29]. A lack of awareness about one’s illness can result in a type of vulnerability, which can in turn cause anxiety about the treatment process. In fact, education based on the self-regulation model can raise awareness, which can reduce anxiety and concerns [30].

The chronic nature of primary dysmenorrhea, its prevalence, and symptoms are major factors that disrupt quality of life and social activities for female adolescents. There is also a widely understood gender inequality in medical research resulting in a dearth of research on menstruation problems and women’s health issues more generally [31]. Therefore, this study will aim to investigate the effects of psychoeducational intervention using the Leventhal self-regulation model during menstrual distress in adolescent girls with primary dysmenorrhea.

## Methods

### Study design and participants

**Fig. 1:**
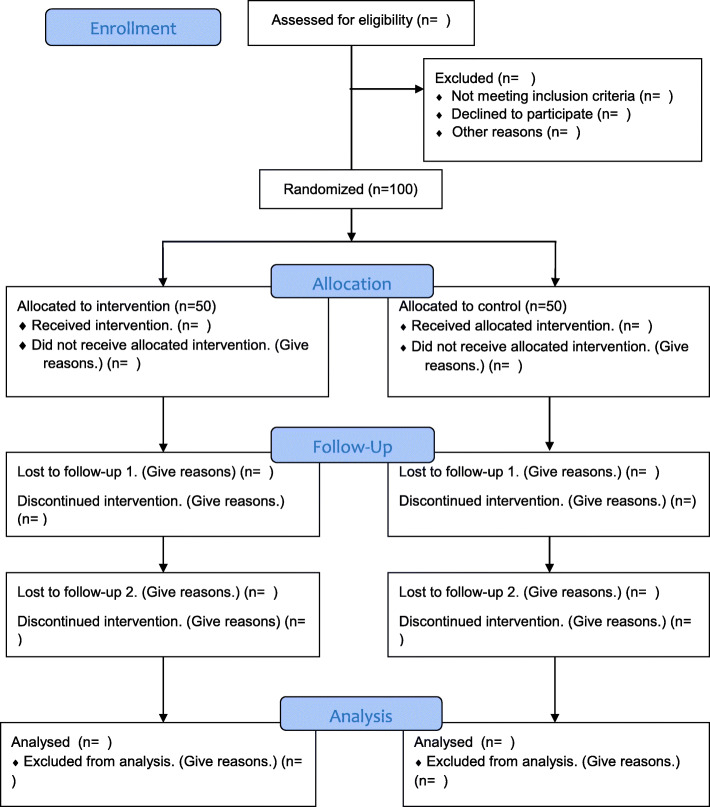
CONSORT flow diagram

The design of this randomized controlled trial is designed based on standards devised by the Consolidated Standards of Reporting Trials (CONSORT) (Fig. 1). Adolescent girls aged 14–19, attending high schools in Qazvin City, and experiencing moderate to severe pain (pain intensity ≥ 4 based on the participant’s VAS score for two consecutive months in a preliminary screening) will be recruited to the study. Inclusion criteria will include having regular menstruation and experiencing menstruation for at least 2 years. Girls will be excluded from the study if they have experienced secondary dysmenorrhea due to endometriosis, adenomyosis, subacute endometritis, pelvic inflammatory disease, intrauterine devices, ovarian cysts; congenital anomalies of the pelvis and stenosis; history of gynecological surgery; or report a history of mental or physical illnesses or substance abuse (based on their self-report and student’s health reports). Participants will also be excluded if they are married or have taken specific medications for premenstrual syndrome (e.g., fluoxetine) in the past 6 months.

### Sample size estimation

The estimation of the sample size is based on the study of Wong et al. considering the mean menstrual distress score for intervention group 23.96 ± 4.79 and 26.61 ± 5.1 in the control group [32], alpha = 0.05, and *β* = 0.2; the estimated sample size in each group will be 51 individuals in each group. Peng et al. suggested adding a 20% attrition rate for youth, school-based studies [33], so the estimated sample size for the current study will be 60 individuals for each group. As we have two groups, the total sample will be 120 female students.

### Sampling and randomization

Given the likelihood of information sharing between the students at the same school, the intervention and control groups will be selected from separate schools. A two-stage cluster sampling design will be used to selected study samples. At the first stage, twelve schools will be randomly selected from a list of eligible schools (girls-specific schools and not involved in similar educational intervention) in Qazvin city using a computer-generated random number. The list of schools will be provided by the department for Education in Qazvin. At the second stage, one class will be randomly selected from each school through a simple random method. Ten eligible individuals will be selected using random sampling method. Assigning of schools to the control and intervention groups will be done via random sampling method. Therefore, six schools will be assigned to the intervention, and six schools will be assigned to the control group.

### Intervention

#### Intervention sessions

The sessions will be between 60 and 90 min apiece, and they will run for three consecutive weeks (one session per week). They will be held in groups consisting of 8–10 people [34]. In general, 5 intervention groups will be formed, and 15 educational sessions will be held at the school within 2 months of the sessions. If needed, individual counseling sessions on menstrual distress and common problems for adolescents will also be held.

#### Intervention details

The design of the intervention will be based on the Leventhal self-regulation model [30]. This model seeks to improve the perception of disease by increasing adolescents’ awareness of their menstruation distress, as well as the control of their emotional responses and coping behavior. For this purpose, hypotheses about the self-regulation model will be used to design the training program. A self-regulated paradigm is a conscious personal management system that involves the process of guiding one’s thoughts, behaviors, and emotions to achieve goals. Therefore, the educational material used for adolescent girls will be designed to be performed in three sessions:

The first session will be based on five dimensions of illness perception [35]. This stage is broken into four steps. The first step involves assessing the adolescent’s perception of the nature of menstrual distress. This assessment includes psychological symptoms related to menstrual distress (e.g., anxiety and depression) and physical symptoms (e.g., fatigue, pain, and weakness). It also examines the beliefs of adolescents about the prevalence of menstrual distress and its complications (e.g., beliefs related to the identity of the disease). The second step will involve assessing the girls’ perceptions about the cause of the menstrual distress and their beliefs about menstrual distress, in order to eliminate their untrue beliefs about the causes of this disease. Then, the duration of the illness and the adolescent’s perception of the duration of the illness will be examined (i.e., the timeline). The third step will be concerned with any distress menstruation has on the adolescent’s life (i.e., the consequences). During the last step, the researchers will examine the adolescent’s perception of the efficacy of existing treatments, in order to control and improve the severity of menstrual pain and relieve symptoms of menstrual distress (e.g., ideas about treatment and improvement). This session will include techniques for self-control, verbal encouragement, feedback, behavioral assessment, and ways to successfully control their illness experiences.

After reviewing the first session, the second session will begin. The adolescents will be asked to talk about their feelings and ambiguities about menstrual distress, and their beliefs and knowledge challenged by the researcher. The researchers will provide information to participants relating to how they could change their misperceptions about the disease. They will be asked if they had any questions about the previous session. In the last 30 min of each session, stress and relaxation strategies will be taught, and the adolescents will be asked to practice these techniques.

The third session will involve an evaluation and closure of the sessions. Beforehand, the adolescents will be notified that this session would be the last one. After they arrive, the content of the previous sessions will be reviewed. Then, they will be asked about the impact of new training and experiences on their lives. Problems and obstacles for each adolescent will be explored. Exercises from the previous sessions will be repeated, and their questions answered. Different methods will be used to increase the effectiveness of the training program.

To ensure adolescents are provided sufficient information to relate to the cognitive and psychological dimensions, a booklet will be used, in addition to face-to-face instructional education. The booklet will aim to improve illness perceptions by providing simple illustrations with explanations. The educational content of this booklet will identify and define menstrual distress, provide a brief explanation of misconceptions about the menstrual cycle, and offer activities authorized and unauthorized during the course and instructions about personal hygiene, physical activity, exercise, nutrition, stress-control techniques, and relaxation. The qualitative content of the booklet will be reviewed by ten faculty members. At the end of the three sessions, this booklet will be given to the intervention group. In addition, if participants had any further questions about the study or illness, the researcher’s contact information will be provided to the adolescents.

### Data collection tools

The following four tools will be used to collect data in the present study:

1. *Demographic and menstrual questionnaire*: This tool will collect data about demographics related to menstrual problems, including age, age of menarche, education level, field of study, marital status, characteristics of the menstrual cycle (e.g., menstrual period and duration of bleeding), pain medication history, drug addiction, height, weight, and body mass index. It will also collect data about the adolescents’ histories of dysmenorrhea or menstrual distress (e.g., in the mother or sister of the adolescent), secondary dysmenorrhea, underlying diseases, and vaginal sex. This questionnaire will be developed based on the aims of the study, and its validity will be evaluated using the content-validity method by the faculty members of midwifery at Qazvin University of Medical Sciences.
2. *Visual analog scale (VAS*): This valid, reliable tool will be used to assess the severity of dysmenorrhea [36]. The tool is a 100-mm ruler, with zero at one end (which indicates complete pain relief) and ten on the other end (which indicated the most severe pain imaginable). Participants will mark the severity of pain on the ruler. This scale is commonly used in pain assessment, due to its ease of use [37].
3. *Moos Menstrual Distress Questionnaire*: This tool was designed by Radolf Moos [38] to investigate the impact of menstrual distress on one’s daily activities. Its internal correlation is reported to be 83% for pain and 94% for autonomic activity. It consists of 47 questions, which are divided into 8 subgroups. It measures the patient’s general complaints about various types of symptoms during menstruation. Participants will be asked to report symptoms experienced during menstruation, according to the Likert scoring system. A score of 1 for a condition will indicate no experience of any symptom, and 4 will suggest the most severe experience (i.e., almost debilitating). The short form of this questionnaire consisted of 16 to 19 questions. It was widely used to examine physical and psychological symptoms during the premenstrual, intra-menstrual, and intermenstrual phases in various contexts [22]. The validity and reliability of the Farsi version of this questionnaire were evaluated by Qorbanalipour et al. [39]. Therefore, it can be used in various studies for therapeutic purposes in the future.
4. *Illness Perceptions Questionnaire*: The IPQ was first developed by Weinman et al., based on the Leventhal model [40]. Its brief, 9-item, version will be used to assess emotional and cognitive perceptions about the disease which was modified to menstrual distress in this study. The first five questions examine the cognitive embodiment of the disease, including outcome, duration, individual control, treatment control, and nature. Two of the questions relate to affective visualization, including anxiety and emotional response. Scores range from 0 to 10, with higher scores indicating more severe misperceptions about the disease. The validity and reliability of the Farsi version of this questionnaire was confirmed by Bazazian and Beheshti.

### Primary outcome

The primary outcome is the perceived menstrual distress of adolescent girls.

### Secondary outcomes

The secondary outcomes are the illness perceptions and severity of dysmenorrhea pain.

### Procedure

**Fig. 2:**
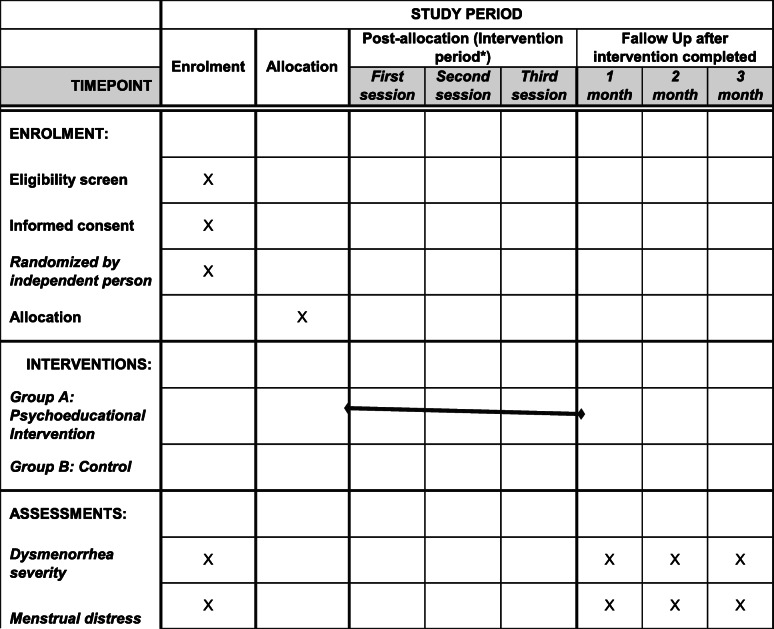
SPIRIT schedule of enrolment, interventions, and follow-up assessments

After obtaining the necessary permission to conduct the study, the researcher will attend the selected schools to identify eligible subjects and invite them to participate. The researchers will provide necessary explanations about the purpose of the research and ensure that data remains confidential. Informed consent will be obtained from eligible subjects. It will be explained that participants can withdraw from the research whenever they chose to. For two consecutive months prior to the intervention, an initial assessment of the severity of dysmenorrhea pain and menstrual distress will be performed in the groups. Questionnaires will be completed by the groups within 3 months after the intervention. Data will be collected using paper file storage. No intervention in the control group will be performed, but the educational content after the end of the study will be made available to the control group participants. If needed, the participants in both groups will be able to contact the researcher at any time. A small gift (a set of six colored pens) will be provided to participants to thank them for their participation. Figure 2 shows the timing of outcome measurement and intervention based on the SPIRIT table.

### Intervention fidelity

The researcher (SA) is responsible for conducting the intervention and holding education sessions. At the time of the study, she is a midwife (BSc) and postgraduate student of counseling in midwifery. Therefore, she has sufficient experience in the field of research, and she has the necessary knowledge to conduct education and consulting sessions. Also, the educational sessions will be randomly evaluated by group instructors (NB and MAS) in 20% of the sessions. Specifically, initial meetings will be held to ensure the researcher’s ability to execute the educational sessions.

### Data management

The researcher (SA) will be responsible for monitoring the data collection process throughout the study process. Data entry into the SPSS software will be controlled by ZA and NB.

### Data analysis method

Collected data will be analyzed on an intention-to-treat basis using MLwin 2.27 [41]. ICC will be calculated within groups and a three-level mixed model will be used with adolescents girls nested in schools. The significance level will be *p* < 0.05. Finally, power of study will be calculated too.

## Discussion

This study will be the first randomized controlled trial to treat menstrual distress in adolescent girls that utilizes a psychoeducational intervention based on the Leventhal self-regulation model. The study results could provide insights into the effectiveness of using this model to treat menstrual distress in adolescents. Other interventions have focused on the effectiveness of reducing the severity of menstrual cramps using yoga [42], hypnosis [43], and the education of self-care behavior [44], for example. However, few other studies have been conducted on this topic, and no interventions have been performed to reduce menstrual distress, in addition to reducing the severity of menstrual pain. Given the high prevalence and impact of the physical and psychological complications of menstrual distress on adolescent academic performance, more interventions are still needed. Fortunately, education based on the Leventhal model of self-regulation is uncomplicated, low-cost, and easy to use. This model has also been used to reduce psychological distress, perceived stress, and anxiety in individuals with chronic diseases, and it has shown beneficial effects [45, 46]. If this intervention shows promising effects, it can reduce the rate of school abstinence and increase student’s academic achievements. More specifically, this study will be designed to focus on the chronic nature of dysmenorrhea and shape the specific understanding of how to manage menstrual distress by using the self-regulation model.

## Data Availability

After performing the main study, the analyzed data and materials will be de-identified and published.

## Abbreviations

CONSORT: Consolidated Standards of Reporting Trials
IPQ: Illness Perceptions Questionnaire
VAS: Visual analog scale
